# Developing a platform for secretion of biomolecules in *Mycoplasma feriruminatoris*

**DOI:** 10.1186/s12934-024-02392-3

**Published:** 2024-04-30

**Authors:** Javier Gonzalez-de-Miguel, Ariadna Montero-Blay, Ludovica Ciampi, Irene Rodriguez-Arce, Luis Serrano

**Affiliations:** 1 Centre for Genomic Regulation (CRG), The Barcelona Institute of Science and Technology, Dr Aiguader 88, Barcelona, 08003 Spain; 2 Orikine Bio, Dr Aiguader 88, Barcelona, 08003 Spain; 3 Universitat Pompeu Fabra (UPF), Barcelona, Spain; 4 ICREA, Pg. Lluis Companys 23, Barcelona, 08010 Spain

## Abstract

**Background:**

Having a simple and fast dividing organism capable of producing and exposing at its surface or secreting functional complex biomolecules with disulphide bridges is of great interest. The mycoplasma bacterial genus offers a set of relevant properties that make it an interesting chassis for such purposes, the main one being the absence of a cell wall. However, due to their slow growth, they have rarely been considered as a potential platform in this respect. This notion may be challenged with the recent discovery of *Mycoplasma feriruminatoris*, a species with a dividing time close to that of common microbial workhorses. So far, no tools for heterologous protein expression nor secretion have been described for it.

**Results:**

The work presented here develops the fast-dividing *M. feriruminatoris* as a tool for secreting functional biomolecules of therapeutic interest that could be used for screening functional mutants as well as potentially for protein-protein interactions. Based on RNAseq, quantitative proteomics and promoter sequence comparison we have rationally designed optimal promoter sequences. Then, using *in silico* analysis, we have identified putative secretion signals that we validated using a luminescent reporter. The potential of the resulting secretion cassette has been shown with set of active clinically relevant proteins (interleukins and nanobodies).

**Conclusions:**

We have engineered *Mycoplasma feriruminatoris* for producing and secreting functional proteins of medical interest.

**Supplementary Information:**

The online version contains supplementary material available at 10.1186/s12934-024-02392-3.

## Introduction

The medical industry is highly interested in the screening of biomolecules with an optimised affinity for their targets [1]. The process of screening for these interactions is often initially done in microorganisms (bacteria, yeast) due to their easy genetic manipulation and reduced costs [2, 3]. Extensive work has been performed on characterising and designing expression cassettes for these species. These include promoters to ensure abundant expression without placing metabolic stress on the chassis organism nor having toxic accumulation levels inside the cell [4]. Ideally, the protein of interest should be exposed at the surface (for interaction screening) or secreted (for functional analysis) via the addition of a transmembrane segment to their N-terminal or a signal peptide, respectively. Secretion favours a simpler purification process [5], which might become relevant when upscaling the use of the host chassis to industrial levels.

The mycoplasma genus has a set of unique traits that make it an interesting chassis to develop screening methods. They are a part of the Mollicutes class whose most characteristic traits are possessing the smallest reported genome sizes (0.5–1.3 Mbps) in bacteria that can be grown in the lab and not having a cell wall [6]. For their apparent simplicity, they have long attracted the attention of the systems and synthetic biology community. They were first studied as a model with which to define the essential genes to sustain life [7]. These studies led to their use as the template from which minimal synthetic genomes and cells could be chemically synthesised [8, 9]. More importantly, secretion in most used bacteria requires additional sorting through a thick peptidoglycan cell-wall (Gram-positive) or through the periplasmic space and a further outer membrane (Gram-negative) [10]. This further step does not exist in mycoplasma, potentially ensuring homogenous secretion of all the screened variants as has been shown in *Mycoplasma pneumoniae* (*Mpn*) [11–13].

A recent application for a species of this genus has come in the medical synthetic biology field, where the capacity of the model organism *Mpn* to infect lung tissue has been harnessed to developed therapies against infectious diseases and inflammation caused by both Gram negative and positive bacteria [11–13]. In these studies, *Mpn* was attenuated and made to express heterologous proteins with bactericidal and biofilm degradation capacities [13]. *Mpn* was also shown to express and secrete a fully folded human and active interleukin (IL)-10 [13], despite this molecule’s folding depending on the correct formation of disulphide bridges [14]. However, the level of expression was too low for obtaining a biological effect. This was solved by engineering a single chain IL-10, increasing its biological activity fifty times and improving its expression by four times [13].

*Mpn* is an excellent chassis for lung therapy but has a main significant roadblock: the slow dividing time of most species (8 to 20 h.) compared with for example *E. coli* (30 min.) or yeast (1.5 h) [6]. Recently, a new Mycoplasma fast-growing species, known as *Mycoplasma feriruminatoris* (*Mfr*), has been characterised [15]. It has an estimated doubling time of 27–33 min., comparable to that of industrially relevant species [15, 16]. It has also proved amenable to extensive genome manipulation [16]. As part of the ruminant-infecting mycoplasma mycoides cluster, it has already been proposed as a chassis for veterinarian vaccine design [16]. Combining the fast growth of *Mfr* with the relative simplicity of protein secretion in the *Mycoplasma* species makes it a suitable chassis for testing the functionality of engineered proteins of interest to be expressed in *Mpn.* It also opens the possibility to develop other future applications like protein interaction screening methods. However, so far there has been no description of neither promoter nor secretion signals in *Mfr*, except for the SynMyco promoter [17]. The SynMyco mycoplasma universal promoter is based on a multiple sequence alignment of the highly expressed *tuf* gene of several mycoplasma species to identify common elements. Its functionality capacity as a strong promoter had been tested in a modified version of the Tn4001 expression vector widely used for random chromosomal insertion in the mycoplasma field [18]. The SynMyco promoter demonstrated higher transformation efficiencies in different Mycoplasma species when using it for expression of both the transposase and the antibiotic resistance gene [17]. However, this promoter was not tested or optimised for *Mfr* specifically.

The lack of biological tools on *Mfr* also applies to secretion signal peptides. All mycoplasma species use the Sec secretion pathway [10]. This highly conserved pathway relies on N-terminal signal peptides which drive translocation, insertion, and cleavage from the membrane. Signal peptides from this pathway have three well-defined regions: a positively charged region following the start codon, a hydrophobic domain which will form a transmembrane helix, and a polar region containing a cleavage site to be recognized by a membrane protease known as a signal peptidase (SPase) [19, 20].

In this work, we sought to develop a platform for optimized expression and secretion in *Mfr* for testing biomolecules. To do so, we first identified highly active promoters and rationally implemented mutations to improve their activity. Then, putative secretion peptides were uncovered using *in silico* tools and validated experimentally in a luminescence-based assay. Finally, we tested its ability to express heterologous proteins with a selection of clinically relevant molecules, including IL-1β antagonist Isunakinra [21], human IL-22 and nanobodies for murine PDL1 and CTLA4.

## Results

### Identifying the best promoters in *Mfr*

**Fig. 1:**
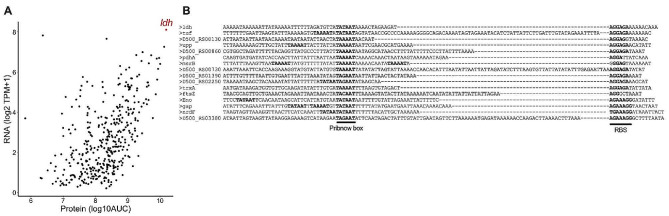
Analysis of promoter regions in*M. feriruminatoris*. (**A**) *Mfr* was grown to exponential phase and its transcriptomes and proteomes were measured. The former is quantified in log2 (TPM + 1) and the latter in Area Under Curve (AUC). The most expressed gene corresponding with lactate dehydrogenase (*ldh)* is shown in red. Both analyses were performed on biological replicates (*N* = 2). Raw data of both replicates are included in Suppl. File 1 and 2. (**B**) Analysis of the top 5% promoters in *Mfr.* They are shown with the main features (Pribnow box and RBS) sequence highlighted in bold. Promotes inside an operon are excluded

The first step towards building an optimised platform for protein screening in *Mfr* is to characterise optimal promoter regions. For this, both the transcriptome and proteome of *Mfr* were analysed using RNA-seq and Mass Spectrometry (MS), respectively (Fig. 1A, Suppl. File 1 and 2). The proteomic analysis includes both data from the cell and from the proteins secreted into the growth culture in vitro. Correlation analysis between proteome and transcriptome revealed the lactate dehydrogenase (*ldh*) promoter as the top candidate having both optimal protein expression and production (Fig. 1A).

We then looked at the top 5% expressed genes considering as potential promoters the DNA region up to 80 bases upstream of the start codon. We did not consider those genes that were located inside an operon. Analysing the selected promoter regions revealed some key features controlling gene expression in *Mfr* (Fig. 1B). As observed for other Mycoplasma species, the − 35 box motif was mostly absent and seemed to have lost its importance throughout some species in this genus [22]. The Pribnow box motif was found in all the promoters analysed, and in many instances more than one Pribnow box was found in the same putative promoter. An extended Pribnow box motif (TGN) could also be observed in some of the sequences (Fig. 1B) [23]. Almost all first genes of an operon presented a nearly canonical Ribosome Binding Site (RBS) sequence close to the start codon (Fig. 1B) [24].

### Optimising expression in *Mfr*

**Fig. 2:**
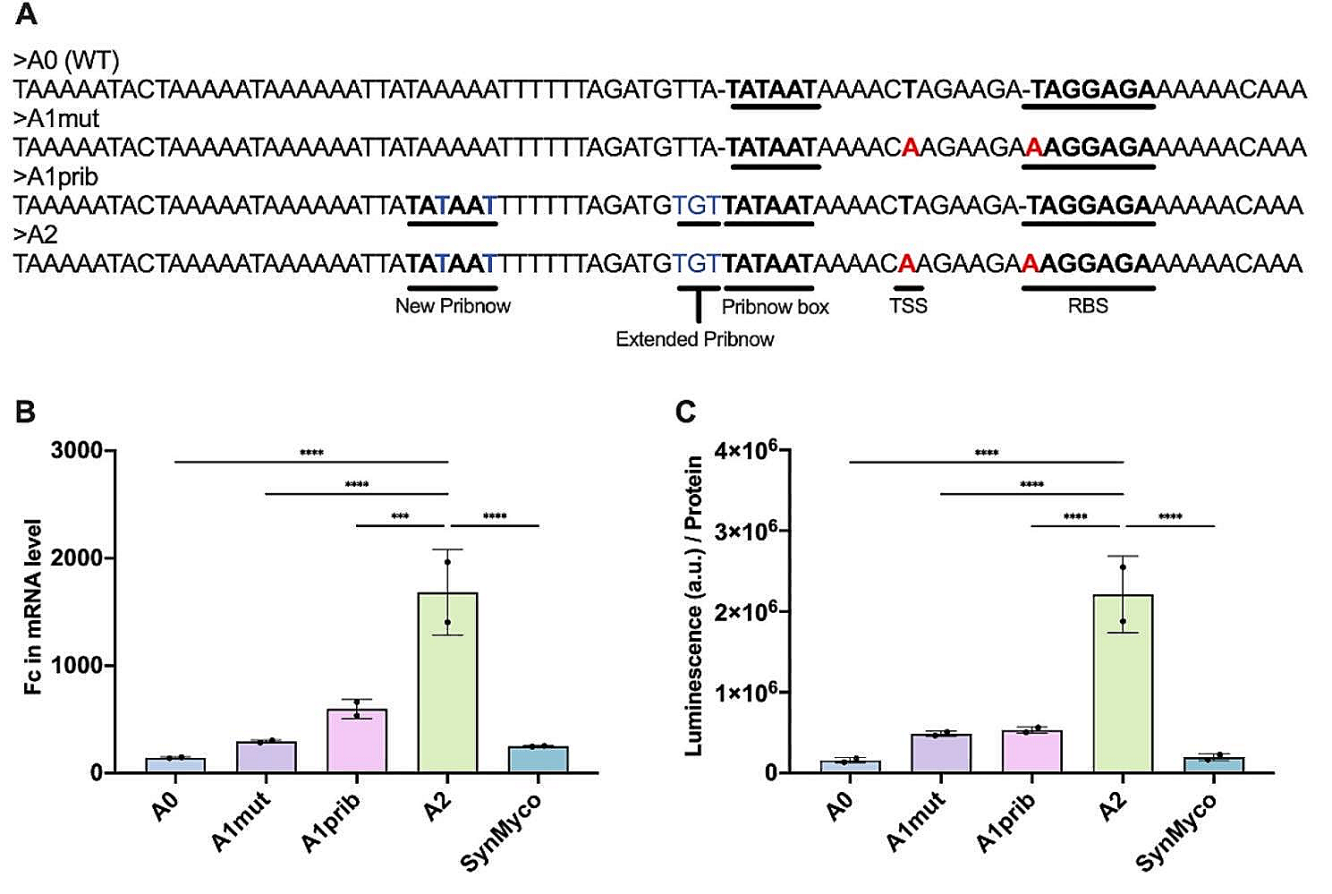
Rational design of *Mfr* promoters. (**A**) Sequences of tested promoters with key features highlighted in bold. (**B**) Gene transcription analysis of *Nluc* reporter gene cloned downstream of promoters A0, A1mut, A1prib and A2 at exponential phase (20 h). Fold change (Fc) expressed in relation to housekeeping genes *rpsM*, *gapdh* and *gyrA* calculating ΔΔCt (see Methods). This experiment was performed twice with three technical replicates (*N* = 2). Statistical test was performed using One-way ANOVA with multiple comparisons. (**C**) Nluc production measured by luminescence at different time points normalised by total protein concentration determined by BCA. This experiment was performed twice with two technical replicates (*N* = 2). *P*-value: (*, < 0.0332; **, < 0.0021; ***, < 0.0002; ****, < 0.0001). Data is shown as average ± standard deviation (SD)

Based on the above data, we selected the *ldh* promoter for further design. Rationally mutated versions of the WT *ldh* promoter (referred to as A0) were designed following two strategies (Fig. 2A). In the first strategy, the putative Transcription Start Site (TSS) was changed from thymine to adenine, which had been previously described to improve transcription in strong promoters [25] and the RBS sequence was modified to be canonical (AAGGAG) [17], leading to the A1mut promoter. The second strategy consisted of adding a second Pribnow box motif upstream of the native one and incorporating an optimal extended Pribnow box with an extended Pribnow sequence (TGT) [25], generating the A1prib promoter. Both sets of mutations were combined in the A2 promoter (Fig. 2A). The strength of the different versions was tested via the expression of the Nanoluc luciferase (Nluc) reporter [26], allowing for quantification at both the RNA and protein level. As a positive control, we added the SynMyco promoter [17].

Analysis of Nluc reporter show that the engineered promoters increased RNA and protein expression in comparison with the wild-type A0 promoter, with the A2 promoter having the highest transcription efficiency (Fig. 2B) and protein production (Fig. 2C). High expression of a recombinant protein can lead to growth defects due to toxic accumulation in the cytoplasm or a metabolic burden effect [27]. This was not the case for the A2 promoter when compared to the reference strain expressing only the antibiotic resistance (Suppl. Figure 1). Unless stated, all further experiments in this work have been performed using the A2 promoter.

### Identification of secretion signals in *Mfr*

To identify putative secretion signals we followed a two-step strategy. On one hand, we did free label MS quantification of the cell proteome and secretome, identifying ten proteins in the cell supernatant (Table 1, Suppl. File 2). On the other hand, we analysed the entire predicted proteome of *Mfr* (848 ORFs) *in silico* using the SignalP 6.0 software [20, 28, 29]. Twelve signal peptides were predicted with a score above 0.48 (Table 1).

**Table 1:** *Mfr* secretion signals identified by SignalP 6.0 server. All *Mfr* ORFs were evaluated. Signal sequences are shown divided by their three characteristic regions: the positively charged, the hydrophobic transmembrane and the polar at the C-terminal. The added residues after the post-cleavage sites also appear. Tested sequences are in bold. Lipobox motifs appear underlined. Membrane anchor means that there is a predicted C-terminal membrane helix that could anchor the protein after cleavage of the signal peptide. Suppl. File 2 summarises the AUC for the secreted proteins

# ID	SP (Sec/SPI)	Signal Peptide			Post-cleavage added sequence	Secretome (MS)	Membrane Anchor/Lipobox
		Positive charge	Hydrophobic transmembrane	C-terminal			
s55	0.85	MKK	LLTILTTLIGT	SGSISA	VVSCKGG	Yes	Lipobox
s515	0.82	MK	ITAILSSLFLSPTL	LNT	SPILVNGG	No	Membrane Anchor
s1260	0.58	MKK	LLSILAICTLATTSILLSPLL	INNNSNNNIVLKA	ETKKEGG	No	Signal Peptide
s1535	0.54	MNLLKKKKNK	ILAFAILAGLMTSASLGSTVFYSIA	ADNSLA	KDVDSGG	No	Membrane anchor
s1545	0.66	MNLLKKKKNK	ILAFAILAGLMTSASLGSTVFYSI	ADNSLA	KEVDSGG	Yes	Membrane anchor
s1575	0.53	MKINKNHSR	LLKLISIVTITSSSIILPSF	LVT	KNQESGG	No	Membrane anchor
s2165	0.99	MKK	LLTLLTISTLLVI	PTSSS	FLINKGG	No	Signal Peptide
s3040	0.99	MK	LFLPTLFLLSNSITP	SLA	NSVNVVNGG	No	Membrane Anchor
s3290	0.5	MKLVKK	LGFLSLSAISILGPL	AA	INNLTDNNGG	Yes	Membrane Anchor
s3620	0.61	MKK	VLGITLLGSII	ATASA	SVVSCSVGISLDGG	No	Lipobox
s3960	0.48	MKK	LLALLAVTSILT	SSGITY	VIHENGG	Yes	Signal Peptide
s3965	0.78	MKK	LLAVLIGLTLFTT	SGVSYV	AYDNIGG	Yes	Signal Peptide

Five of the secreted proteins in MS were predicted to have a signal peptide (s55, s1545, s3290, s3960 and s3965). Seven of the *in silico* predicted sequences having a signal peptide were not identified in the secretome (s515, s1260, s1535, s1575, s2165, s3040 and s3620). Genes s2165 and s3040 were very poorly expressed at RNA level (Suppl. File 1). For the remaining five, two of them (s1535 and s1575) belong to the well-reported Mycoplasma IgG cleavage system which is attached to the membrane [30], s515 has a C-terminal membrane helix that will anchor the protein to the membrane and s3620 has a putative lipobox downstream of the cleavage site (VVSC) [31]. Finally, s1260 has a low prediction score for a signal peptide (0.58) (Table 1). Having a C-terminal membrane helix for anchoring does not invalidate the use of the signal peptide of the corresponding gene for secretion, therefore we considered all putative secretion signals with a good score, no lipobox, as well as those found in the secretome for further screening.

Based on these results, we selected nine sequences to be tested (s515, s1535, s1545, s1575, s2165, s3040, s3290, s3960, s3965) (Table 1). For these signal peptides at least five residues of the native protein were added after the predicted cleavage site and two glycines were further incorporated to add flexibility to the protein to be cleaved while in the membrane (Table 1). The final sequences were fused to the N-terminus of Nluc reporter construct and expressed under the A2 promoter.

We then determined the luminescence of the cell culture supernatant (Extracellular signal) and of the cellular pellet (Intracellular signal) and calculated the secretion efficiency ratios as Extracellular/Intracellular signal. As a control, A2 expressing NLuc without secretion signal was added (A2_NLuc).

**Fig. 3:**
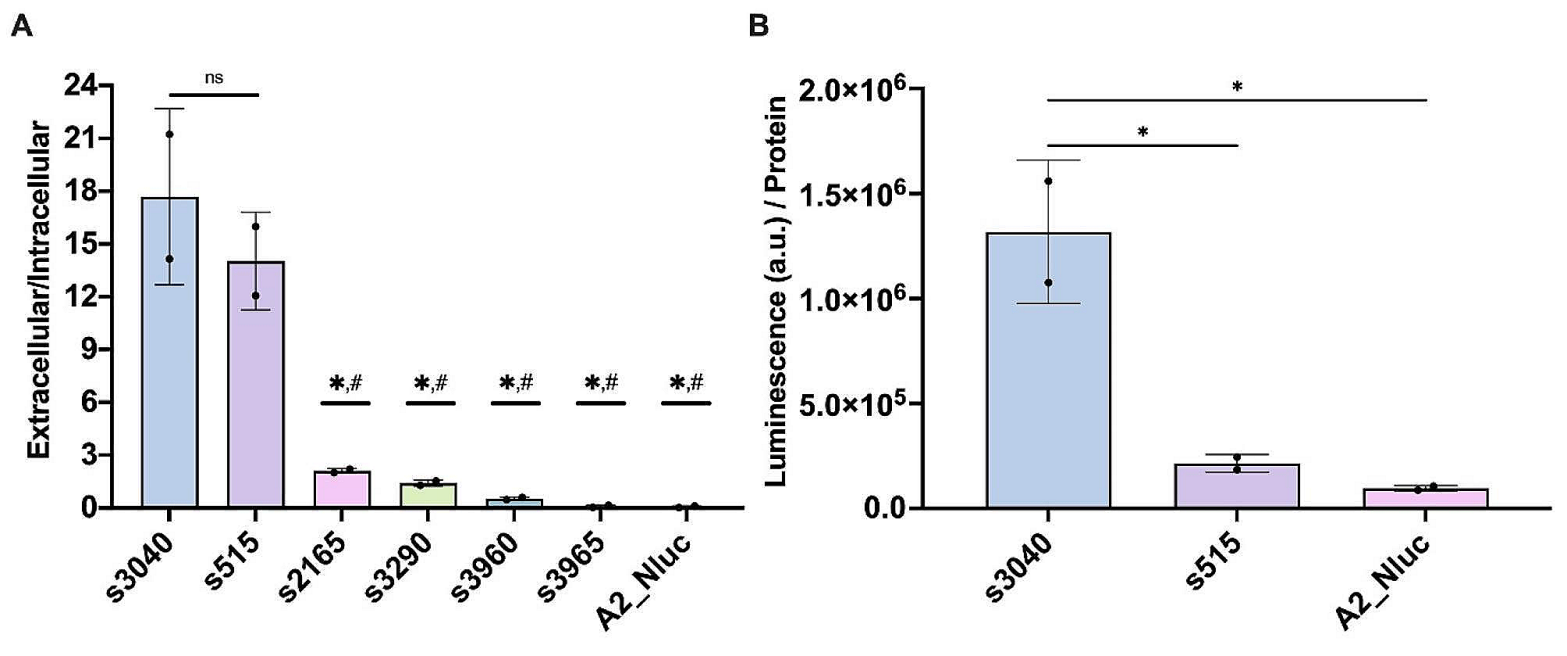
Secretion signal analysis. (**A**) Secretion efficiency of selected signal peptides. *Mfr* strains expressing several signal peptides with a luminescent reporter were grown to exponential phase. Luminescence of both supernatant (extracellular) and pellet (intracellular) was measured to obtain a secretion efficiency ratio. Data are shown as average +/- SD of two biological and technical replicates (*N* = 2). Statistical analysis was performed using One-way ANOVA test of multiple comparisons. The statistical analysis shown both compared to s3040 (*) and s515 (#) (*p*-value < 0.0002). (**B**) Luminescence signal of the supernatant in s3040 and s515. The signal was measured and normalised by quantifying the total protein in the pellet. Data are shown as average +/- SD of two biological and technical replicates (*N* = 2). Statistical analysis was performed using One-way ANOVA test of multiple comparisons (*, *p*-value < 0.05)

Signal peptides s515 and s3040, with SignalP 6.0 scores of 0.82 and 0.9 respectively (Table 1), showed a significantly higher secretion efficiency to the rest (Fig. 3A and Suppl. Figure 3). Interestingly, both secretion signals are found in predicted S41 proteases that have high sequence homology (53%), although the signal peptides themselves show few similarities (Suppl. Figure 4 A). Neither of them was detectable by MS in the secretome of *Mfr* cultures (Suppl. File 2). This may be explained due to the presence of a C-terminal transmembrane helix that will anchor them to the surface of the bacteria, as well as the low expression in the case of s3040 (Suppl. Figure 4B). The signals belonging to the MIB-MIP system were also tested in a similar experiment but gave no significant signal over the negative control (Suppl. Figure 3).

As the overall difference in efficiency for the two best candidates seemed non-significant, the actual production values were used to separate between them. The A2_s3040 platform does show a much higher total protein production (Fig. 3B). In this case, there was a slight effect on the fitness of the bacteria, but not decisively altering growth. (Suppl. Figure 2 A-B). We did a further experiment in a larger culture in experimental conditions closer to that of protein production, and we confirmed a small decrease in cell growth (Suppl. Figure 2 C-D), but not enough to compromise production of the target proteins. Thus, for testing of the expression of proteins with clinical interest in *Mfr*, we used the A2_s3040 platform.

### Expression of heterologous proteins in the *Mfr* platform

To test the versatility of the A2_s3040 platform to secrete proteins, we selected different proteins of medical relevance: hIL-22 [32] (PDB: 14MR); Isunakinra, a clinically approved antagonistic chimera of IL-1β signalling (PDB: 4GAI) [21]; and nanobodies for murine PDL1 (PDB: 5DXW) and CTLA4 (PDB: 5E03) [33]. The proteins selected have different secondary and tertiary structures and, except for Isunakinra, contain disulphide bridges essential for their activity.

**Fig. 4:**
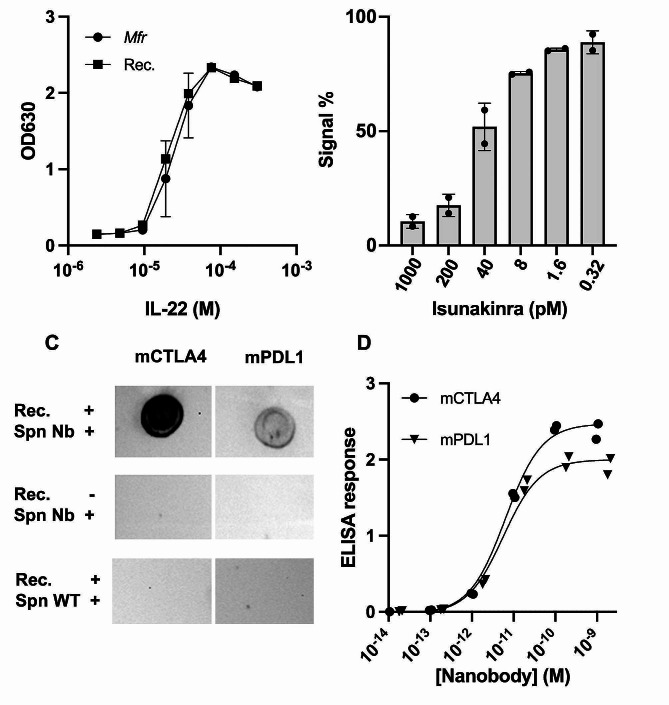
Validation of *Mfr* platform secretion of clinically relevant active biomolecules. (**A**) HekBlue reporter activation measured by absorbance 630 (OD630) of hIL-22 secreted by *Mfr* (circle) compared to commercially recombinant protein produced in *E.coli* (square). Data shown as average +/- SD of two biological replicates with two technical replicas (*N* = 2). (**B**) Antagonist response of increasing concentrations of Isunakinra produced in *Mfr* against HekBlue cell lines stimulated with 9 pM of recombinant IL-1β. Data shown as average +/- SD of two biological replicates with two technical replicates (*N* = 2). (**C**) Dotblot assay measuring the binding of nanobodies produced in *Mfr* against murine CTLA4 and PDL1. Rec. + indicates the addition of the recombinant protein. Spn Nb refers to the addition of the respective nanobody. Spn WT indicates where supernatant of WT protein was added to check for unspecific background to the target protein. The exposure time was 4 min. and 21 s. (**D**) ELISA assay measuring the binding of murine nanobodies to their target. ELISA response refers to the optical density at 450 nm subtracted to the one at 560 nm. Data is shown with each biological replicate individually with a calculated curve for each nanobody (*N* = 2)

Secretion of folded hIL-22 was checked by ELISA and its functionality validated in a HekBlue reporter assay (Fig. 4A). The average production of folded molecule was 155 ng/ml. There was no significant difference observed between the EC-50 (2*10^− 5^ M) values of the secreted protein and the commercially available protein produced in *E. coli* (1.5*10^− 5^ M) (Rec). The secretion of Isunakinra was confirmed by using the Hibit luminescent tag [34] and its activity tested in a competition assay against IL-1β in HekBlue cells (Fig. 4B). We observed an IC-50 comparable to the previously published [21]. Finally, the secretion and functionality of murine nanobodies capable of recognising their targets (mCTLA4 and mPDL1) was evaluated via a DotBlot assay (Fig. 4C), which shows a specific response to the target recombinant protein compared to the supernatant (Spn) of WT strain. For a more quantitative response, an ELISA experiment was performed to measure the binding affinity of each nanobody to its target (Fig. 4D). The EC50s for the nanobodies against mCTLA4 and mPDL1 were 6.3 and 5.6 pM, respectively.

In all cases, *Mfr* was able to secrete fully active heterologous proteins, confirming its potential as a versatile platform for testing and screening biomolecules with clinical interest.

## Discussion

Mycoplasma are potentially interesting for different biotechnological and medical applications [11–13, 16]. In the case of *M. pneumoniae* we had previously shown that it could be used for lung therapy since it can secrete enzymes and functional complicated biomolecules containing disulphide bridges like IL-10 [13]. This ability, as well as potentially exposing them on the membrane, could be interesting not only for medical applications, but also for other purposes like screening of protein variants [35]. Expression of transmembrane proteins from mammalian species would require adaptation given the very specific lipidic environment of the mycoplasma membrane [36]. However, the slow growth of *Mpn* (8–20 h.) makes it impractical for this purpose. Searching for protein variants could be done in well-established model organisms (*E. coli*, yeast) and then cloned in *Mpn*. However, due to the particularities of mycoplasma (absence of a cell wall and genome simplicity), selected mutants tested in these systems might not be secreted or folded correctly in *Mpn*. There are also few reported protein chaperones (*dnaK*) that could aid with folding [37]. Therefore, it is better to use an organism as close to *Mpn* as possible. *Mycoplasma feriruminatoris* (*Mfr)* could be an optimal candidate as it the mycoplasma with the fastest dividing time (0.5–1 h.) [16].

The optimisation of secretion cassettes has been performed for commonly used bacterial workhorses. In the case of *Mfr*, the work had to be started by selecting and designing optimal promoters as only one had been described in the literature. Using -omics data, we could determine that *Mfr*, like *Mpn*, does not have a clear − 35 element [25]. Unlike in *Mpn*, the RBS is present in the promoter of the first gene of operons and therefore seems to play a key role in protein translation. Based on these analyses, we optimised the natural strongest promoter of lactate dehydrogenase (*ldh*) by either adding point mutations to create a new TSS and an optimal RBS, or an extra Pribnow box and designing an optimal extended endogenous Pribnow. Combining both approaches led to obtaining an improved version of *ldh* (A2 promoter) with an 11x fold increase in transcription rate which was further increased at the protein level (13x).

Obtaining an efficient secretion signal was carried out by combining both *in silico* and experimental approaches. From the experimental data, two candidate secretion signals were clearly above the rest (s515, s3040). Interestingly, both proteins harbouring these signals were found to be S41 proteases with a predicted similar architecture. They consist of a Sec Type I secretion signal with a cleavage site followed a highly conserved sequence [10] and a C-terminal transmembrane domain followed by positively charged residues (Suppl. Fig. 4B). They could not be found in the proteomic data from the supernatant because they remain anchored by their C-terminal helix (supported by their detection in the *Mfr* cellular membrane in other studies) [16]. Such proteases were studied in the phylogenetically similar species *Mycoplasma mycoides subsp. capri* (*Mmc*) [38]. Homologues for 515 and 3040 proteins exist in *Mmc* (MLC_1030 and MLC_3270, respectively). The former shows little presence on the supernatant, while the latter is more abundant in the supernatant fraction (38x) [38]. In this same study a homologue of this protein 3290 in *Mmc* (MLC_2570) was also found almost exclusively in the supernatant (400x) [38]. It is more striking then than its signal peptide showed such modest activity. This could be explained if release to the medium was mediated by a membrane protease recognising a sequence in the corresponding protein as happens with MPN142 in *Mpn* [39], although it has not been identified. Thus, although MS analysis of the medium and the cell pellet could identify those proteins secreted by a bacterium species it is not always the most optimal approach as lowly expressed genes or those with C-terminal transmembrane helices could be missed, or proteins secreted as a result of a specific protease cleavage could be misleading. Finally, it is also possible that a full *in silico* approach could also miss signals, particularly as the software used in this study has not been trained with mycoplasma.

Importantly, increased expression rate did lead to slight growth defects due to metabolic burden, which was further increased when upregulating the secretion machinery. We still consider this effect manageable given the increased protein production that using the A2 promoter has and the streamlining that protein secretion allows for screening. Other steps to increase fitness would include removal of the antibiotic resistance gene.

A further limitation to explore of this system is the abundant need of horse serum (20%) in the growth media used. Chemically-defined media have been defined for other mycoplasma species [40, 41]. Developing such type of media would further reduce the potential background in screening experiments and should be explored for screening of molecules sensitive to the presence of serum.

The capability of mycoplasma to correctly fold mammalian proteins is crucial to their use in biomedical applications. In this study, we have proven the versatility of this platform by validating the activity of secreted proteins with heterologous tertiary structures. There were two interleukins tested: human IL-22 and Isunakinra, an IL-1β antagonist. The former is an α-helical bundle with one disulphide bond, and the latter consists of β-sheets. Nanobodies for murine CTLA4 and PDL1 with a single disulphide bridge each were also shown to be active. In studies using *Mpn*, the same secretion peptide (s142-opt) has been used to successfully express antibacterial proteins, enzymes and engineered variants of human IL-10 [11–13]. It is remarkable that in both Mycoplasma species a single secretion cassette is effective at expressing such different molecules for these species. This might reflect the relatively simple biology of Mycoplasma proving a strength when designing a living system.

## Conclusions

In this study, endogenous and rationally modified promoters for the fast-growing *Mfr* species were first developed and validated using transcriptomic and proteomic data. An enhanced version of the lactate dehydrogenase (*ldh*) promoter (A2) for enhanced expression showed the strongest activity. Moreover, secretion signal peptides based on *in silico* predictions were tested with the Nluc luminescent reporter, and s3040 was the most suitable one. Finally, the versatility of the final platform based on A2 and s3040 combination was confirmed via expressing a range of clinically relevant proteins with different conformations.

## Materials and methods

### Bacterial culture conditions

*Mycoplasma feriruminatoris* (*Mfr*) G5487 was kindly lent by Carole Lartigue [15]. It was grown at in suspension (180 rpm, 37˚C) in Hayflick media. The base was prepared by preparing an 800 ml dilution of 20 g PPLO broth (Difco, 255,420), 30 g HEPES and 25 ml 0.5% phenol red (Sigma). This mix was then supplemented with 200 ml heat-inactivated horse serum (Life Technologies, 26,050,088), 20 ml of 50% glucose (Sigma, G8270) and 1 ml Ampicillin (Amp) to a final concentration of 10 µg/ml. When corresponding, Gentamicin (Gm) antibiotic was added (100 µg/ml). *E.coli* was grown on LB in agar plates and grown in 2x YT media supplemented with Amp (10 µg/ml).

### Plasmids and cloning protocol

Plasmids based on pMTn4001 [17] were assembled with the Gibson method. PCR was performed with Phusion polymerase (Thermo Scientific, F530S). When amplifying the secretion signals from the *Mfr* genome, DNA was extracted from *Mfr* using the MasterPure Complete DNA Purification kit (Lucigen, MC85200). Clones were isolated into NEBVR 5-alpha *E. coli* (New England Biolabs, C2987P) using heat shock protocol. Plasmids were purified using NZY Miniprep kit, (MB01001) following the manufacturer’s instructions.

For *Mfr* transformation, 10 ml cultures were grown to exponential phase (20 h.) and centrifuged at 8,000 rpm at 4˚C for 10 min. The pellet was then washed under the same centrifugation conditions in chilled electroporation buffer (272 mM sucrose, 8 mM HEPES) to be finally resuspended in 500 µl. A mixture of 30 µl electroporation buffer containing 1.5 µg of plasmid was then mixed with 50 µl aliquots of the cell suspension. The mix was transferred to a 0.1 cm electrocuvette and incubated for 20 min. on ice. It was then electroporated at 1250 V/ 25 µF/ 100 Ω and incubated on ice for 15 min. Then, 420 µl Hayflick was added to the cells and the mix was incubated at 37 ˚C for 45 min. before inoculating into a 50 ml Falcon tube containing 10 ml Hayflick supplemented with Gm (100 µg/ml) (Sigma, G1397) for clone selection.

All strains generated on this study were checked by PCR and Sanger sequencing (GATC Biotech). Primers used for cloning are listed in Suppl. File 3 and strains generated in Suppl. File 4.

### Growth curves

For bacterial growth, pre-cultures of 3 ml Hayflick media with 1:1000 inoculates were prepared for each of the tested constructs. After reaching exponential phase, 1 ml was taken of each for biomass estimation through the Pierce BCA Protein Assay kit (Pierce, 23,225). For growth in plates, 1 µg/ml was inoculated in 1 ml of media and split into replicates in a flat transparent 96-well plate (Nunclon, 168,055) and cultured in an Infinite 200 Pro plate reader (Tecan). Growth was observed by measuring the ratios of optical density (OD) at 430/560 nm and 600 nm each hour until stationary phase (32 h).

For growth in an Erlenmeyer, the pre-cultures were prepared and their biomass quantified in the same manner. They were inoculated at 1 µg/ml in a 25 ml culture in a 125 ml Erlenmeyer flask. At each relevant time point (0,8,10,12,14,16,18,20,22, 32,60 h.), 400 µl were taken for measuring protein biomass and colour change. Each aliquot was centrifuged twice at in PBS 1x at 8,000 rpm 4ºC for 5 min. Colour change was determined as before (OD 430/560 nm) and biomass was determined by protein concentration using the Pierce BCA Protein Assay kit (Pierce, 23,225).

### RNA extraction and RT-qPCR

*Mfr* was grown to exponential phase (approx. 20 h) and bacterial pellet obtained by centrifugation at 4ºC, frozen in liquid nitrogen and stored at -80ºC until use. RNA was isolated by using the RNeasy Mini Kit (Qiagen) following manufacturer’s instructions. Total RNA concentrations were measured in a Nanodrop and samples with OD 260/230 ratio higher than 1.8, were used. For promoter strength determination, RT-qPCR was followed. Reverse transcription was performed using 1 µg RNA with SuperScript II Reverse Transcriptase (Invitrogen, 8,064,014). Following this, qPCR was performed with SYBR Premix Ex Taq II and quantitated on a LightCycler 480 System (Roche, 05015243001). Relative changes in mRNA expression were calculated by the ΔΔCt method [43]. Housekeeping genes *gap*, *gyrA*, and *rpsM* [44] were used for data normalization (Suppl. File 3).

### RNA-seq

*Mfr* culture and RNA extraction was carried out as described above. Illumina libraries were generated at the CRG Genomics unit and sequenced in an Illumina NextSeq2000, producing an average of 10 million paired-end 50 nt reads. Processing of sequencing reads was performed as follows. Adapter sequences were trimmed from short paired-end reads by using the SeqPurge tool (version 0.1-478-g3c8651b) [45], keeping trimmed reads with a minimum length of 12. Reads were aligned to the wild-type genome of *Mfr* genome (NCBI Reference Sequence: NZ_CP091032.1) and to the transposon inserts sequences using bowtie2 v. 2.3.5 [46], with parameters values: end-to-end mode, 0 mismatches (-N), seed length of 20 nt (-L), very sensitive mode (-L 20 -D 20 -R 3 -i ‘S,1,0.50’), maximum fragment length 1200 nt (-X), only best alignment reported (-k 0). Alignment files were converted from SAM format to sorted indexed BAM format using samtools v. 1.9 (using htslib 1.9) [47] and sort (GNU coreutils) 8.26. Reads were further filtered by a minimum quality (MAPQ) threshold of 15, keeping only primary and mapped reads, and converted to sorted BEDPE format using samtools and bedtools v2.27.1 [48]. Fragment counts per annotation region were computed using bedtools, with strand specific overlaps with minimum overlap fraction of 0.5 of read length. Finally, strand-specific per-base coverage was computed using bedtools. Gene expression levels of both biological replicates are shown as Transcripts per million (TPM) based on fragment count. Raw data are presented in Suppl. File 1.

### Luminescence-reporter assay

The abundance of Nluc was measured using the Nano-Glo Luciferase system (Promega, N1110). For this experiment, 3 ml cultures of *Mfr* were grown in the previously described conditions until exponential phase. At this point, 1 ml of each culture was taken and centrifuged at 10,000 rpm. The supernatants were kept on ice until testing. The pellets were washed three times and finally resuspended in 1 ml PBS 1x. In both the supernatants and the pellets, 50 µl of samples were mixed with a 50 µl of a substrate + buffer mix (1:50 ratio) in a flat white 96-well plate (Corning, CLS3917). The plates were incubated in the dark for 10 min prior to reading the luminescence at 1000 ms integration time, 50 ms settle time in an Infinite 200 Pro plate reader (Tecan). Luminescence signal (arbitrary units, a.u.) was normalised by measuring the protein concentration in each sample. For this, 200 µl of the pellet resuspension was centrifuged again and resuspended in SDS 1% lysis buffer. They were sonicated using a Bioruptor sonication system (Diagenode) and On/Off cycles of 30 s. each for 10 min., centrifuged and the protein concentration was determined with the Pierce BCA Protein Assay kit.

### Mass-spectometry (MS) analysis

*Mfr* cultures were grown in the conditions described before. The supernatant and pellets were split by centrifugation at 10,000 g for 10 min. The supernatants were filtered twice in a Millex-GV syringe Filter unit 0.22 μm (Millipore, SLGV033R).

The supernatants were concentrated by adding 1 ml to a MWCO 3 K column and centrifuging until a final volume of 200 µl. A 6:1 volume of cold acetone was added to these samples and stored overnight at -20 ˚C. After this, the acetone was removed by centrifugation at 16,000 g for 10 min at 4˚C. The dry pellet was resuspended in a fresh lysis buffer of 6 M Urea and 200 mM ammonium bicarbonate.

The pellets were washed in PBS 1x three times by centrifugation. They were finally resuspended in the same lysis buffer as the supernatants. The lysates were disrupted with a Bioruptor sonication system (Diagenode) using On/Off cycles of 30 s each for 10 min. Protein concentrations were measured using the Pierce BCA Protein Assay kit. Samples were prepared at a final concentration of 1 µg/ml at 10 µl and analysed at the UPF/CRG Proteomic Facility. The chromatographic and MS analysis was performed with previously described methods [13]. Raw data of proteome and secretome are summarized on Suppl. File 2.

### Human IL-22 ELISA

*Mfr* coding for hIL-22 were grown as described until exponential phase. Supernatants were collected after centrifugation, filtered (0.22 μm) and stored at -80ºC until use. The hIL-22 ELISA deluxe detection kit (Biolegend, 434,504) was used for IL-22 determination (pg/ml) according to the manufacturer’s instructions.

### Extracellular HiBit quantification assay

*Mfr* coding for Isunakinra with a HiBit tag [49] were grown as described to exponential phase. The presence of Isunakinra was checked using the Extracellular HiBit quantification assay (Promega, N2420) at a reduced volume following the instructions of the manufacturer. To do that, 10 µl sample was mixed with 10 µl of a mix containing buffer, substrate and LgBit protein in a ratio of 100:2:1) in 384 well black plates. Luminescence was determined in an Infinite 200 Pro plate reader (Tecan) using following the protocol described for NLuc.

### HekBlue reporter cell assay

The activity of hIL-22 was checked in a HekBlue reporter cell line (Invivogen, hkb-il22) following the protocol provided by the manufacturer. Briefly, cells were grown in DMEM (Thermo, 10,569,010) supplemented with FBS 10% (ThermoA5256701), Pen/Strep (1%) (Thermo, 15,140,122) and used as per the manufacturer’s instructions. A volume of 20 µl of the supernatant was mixed with 180 µl HekBlue reporter cells containing 50,000 cells/well, in a 96 well plate, and maintained at 37 ºC for 16 h. After incubation time, 20 µl of cell supernatant was mixed with 180 µl of Quanti-Blue mix (Invivogen, rep-qbs). Optical density at 630 nm (OD 630 nm) was measured after at least 30 min. of incubation at 37˚C.

For antagonist activity of Isunakinra, HekBlue cells for detection of IL-1β (Invivogen, hkb-il1bv2) were grown as described. Cells were seeded at 500,000 cells/ml in 100 µl and mixed with 50 µl of recombinant IL-1β (Peprotech, 200-01B) (Final molarity: 9 pM) and 50 µl of decreasing concentrations of *Mfr-*produced Isunakinra (starting at 1000 pM) in a 96-well plate. Cells were incubated for 16 h. and the activity of IL-1β measured determined as described for hIL-22. The EC-50 of *Mfr*-secreted hIL-22 was calculated in Graphpad software, using a non-linear regression curve fit ([Agonist] vs. Response, 3 parameters). The IC-50 of *Mfr*-secreted Isunakinra was determined using the same analysis in antagonist mode ([Antagonist] vs. Response, 3 parameters).

### DotBlot assay

Recombinant protein of murine PDL1 (SinoBiological, 50,010-M08H) or CTLA4 (SinoBiological, 50,503-M08H) were bound to a nitrocellulose membrane (Sigma, GE10600001) in 10 µl drops containing 0.25 µg of protein (25 µg/ml). The membrane was then blocked with 5% skim milk diluted in TBS-Tween 0,1% (TBS-T) for 1 h. at room temperature. Filtered supernatants of *Mfr* were then applied to their respective spots on the membrane for 2 h. at room temperature. Then, anti-Hibit primary antibody (1:1000) (Promega, N7200) was then applied diluted in 3% skim milk TBS-T for either 1 h. at room temperature. After washing with TBS-T three times, the secondary antibody (Sigma, A6782) was applied in a 1:10000 dilution for 1 h. at room temperature. The Femto substrate mix (Thermo, 34,094) was used to reveal the signal on an iBright CL 1500 equipment (Thermo).

### Nanobody ELISA assay

Recombinant protein of murine PDL1 (SinoBiological, 50,010-M08H) or CTLA4 (SinoBiological, 50,503-M08H) were diluted in PBS 1x and applied in 100 µl/well to a 96-well Nunc Maxisorp plate (Invitrogen, 44-2404-21). The Wash Buffer used was PBS-Tween 0.05%. Unless specified, each well was washed between every step with at least 250 µl Wash Buffer four times per wash. After overnight incubation at 4˚C, the wells were blocked with 200 µl/well of PBS-FBS 10% 1 h at room temperature. All further incubations, including this one, were done in gentle shaking conditions. This buffer was used for all subsequent dilutions. The supernatants of mycoplasma secreting nanobodies or the appropriate controls were added in serial dilutions. After removal, antibody against HiBit (Promega, N7200) at 1:500 dilution was added at 100 µl/well for 1 h. at room temperature. Secondary antibody anti-mouse IgG produced in sheep (Merck, A6782) was then applied at room temperature for 1 h. After this, substrate peroxidase substrate mix (Biolegend, 42,101) was prepared following manufacturer’s instructions and added at 100 µl/well. The reaction was carried on for 15 min. and stopped with 100 µl of Stop Solution (Biolegend, 77,316) added without washing. The plates were read in no less than 10 min. by measuring the absorbance at 450 and 570 nm and subtracting them. The EC50s were estimated using the GraphPad Prism software in-built analysis of non-linear regression for curve fitting.

### Statistical analyses

All statistical analyses were performed using the GraphPad Prism software and are specified on the figure legend. In all cases, *p* values < 0.05 were considered as statistical difference and the exact value is included in the figure legend.

### Electronic supplementary material

Below is the link to the electronic supplementary material.

Supplementary Material 1

Supplementary Material 2

Supplementary Material 3

Supplementary Material 4

Supplementary Material 5

## Data Availability

The datasets generated during this study are available within the study. The transcriptomic data was uploaded to the GEO database under the accession number GSE245952.

## Abbreviations

Amp: Ampicillin
a.u.: Arbitrary units
AUC: Area Under Curve
BCA: Bicinchoninic Acid
Ct: Cycle threshold
EC: 50-half maximal Effective Concentration
ELISA: Enzyme-Linked ImmunoSorbent Assay
FBS: Fetal Bovine Serum
Gm: Gentamicin
H.: Hour
HS: Horse Serum
IC: 50-half maximal Inhibitory Concentration
IL: Interleukin
LB: Luria Bertani
*ldh*: Lactate dehydrogenase
MS: Mass Spectrometry
Mbps: Mega base pairs
MIB: Mycoplasma Immunoglobin Binding
MIP: Mycoplasma Immunoglobin Protease
*Mmc*: *Mycoplasma mycoides subspecies capri*
*Mfr*: *Mycoplasma feriruminatoris*
*Mpn*: *Mycoplasma pneumoniae*
mCTLA4: Murine Cytotoxic T-Lymphocyte Associated protein 4
Min.: Minute
MWCO: Molecular Weight Cut-Off
mPDL1: Murine Programmed Death-Ligand 1
Nluc: NanoLuc
OD: Optical Density
ORF: Opening Reading Frame
PBS: Phosphate Buffered Saline
PCR: Polymerase Chain Reaction
PPLO: PleuroPneumoniae-Like Organism
RBS: Ribosome Binding Site
Rpm: Revolutions per minute
RT: qPCR-Reverse Transcription quantitative PCR
S.: Second
SD: Standard Deviation
SPase: Signal Peptidase
Suppl.: Supplementary
TBS: Tris-Buffered Saline
TPM: Transcript per million
TSS: Transcription Start Site
*tuf*: Elongation Factor Tu
WT: Wild Type
2x YT - 2x: Yeast extract Tryptone
