# Supplementary material for: Developing a platform for secretion of biomolecules in Mycoplasma feriruminatoris
Source: Microb Cell Fact. 2024 Apr 30;23:124. doi: 10.1186/s12934-024-02392-3 (PMC11059754; doi:10.1186/s12934-024-02392-3)
Supplement: Supplementary file 5 — Supplementary Material 5 [file 12934_2024_2392_MOESM5_ESM.docx]

**Additional Information**


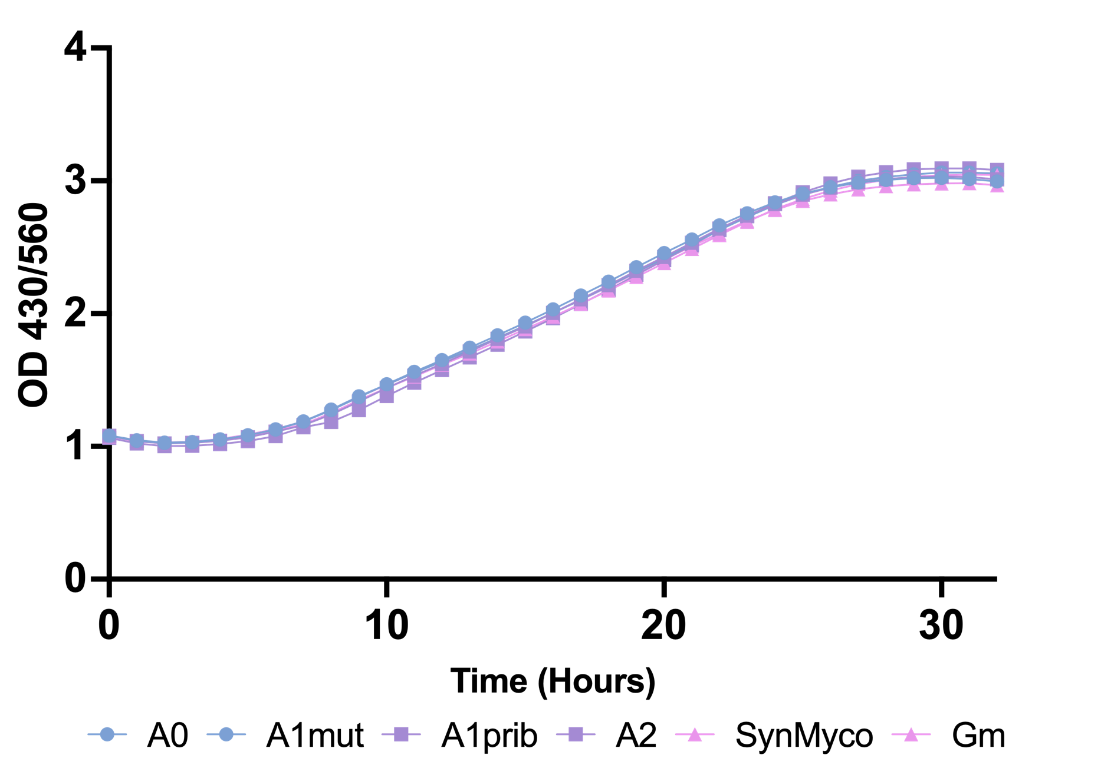


**Supplementary Figure 1. Growth curves of screened promoter clones**. Clones expressing Nluc under the A0, A1mut, A1prib, A2 promoters and SynMyco were grown to exponential phase along with a control clone expressing only the antibiotic resistance (Gm). Colour change was normalised over non-inoculated media. The experiment was performed once in technical and biological duplicates (N = 2) Data is plotted as an average.

**
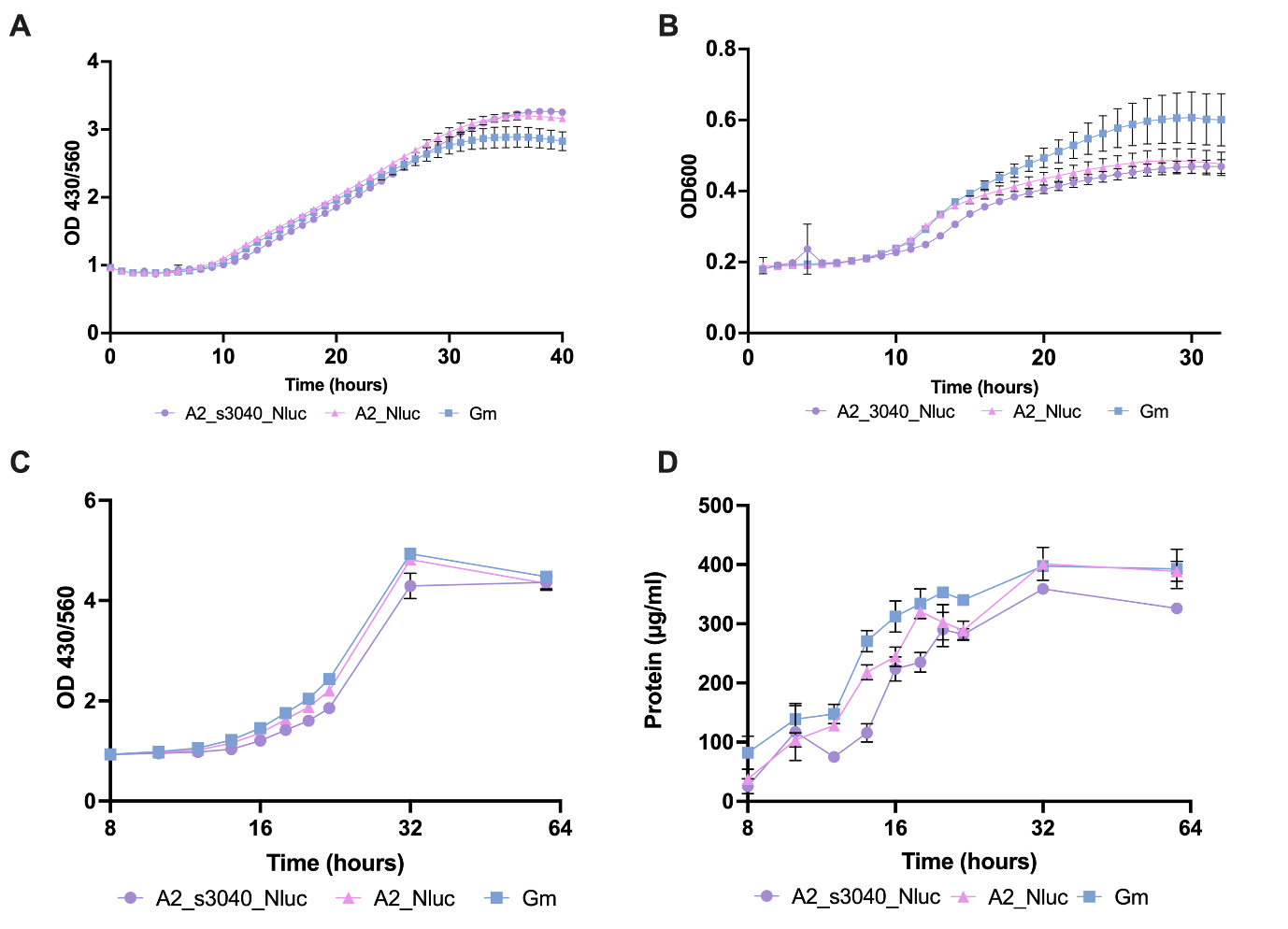
**

**Supplementary Figure 2 Growth curves of *Mfr* strains expressing Nluc under the A2 promoter with and without the secretion signal. (A-B)** Plate growth characterisation. Their growth was measured by observing the change in pH with phenol-red (ratio of OD at 430 and 560 nm), OD (430/560) (A) and OD600 (B). Colour change was normalised over non-inoculated media. Experiments were performed once in triplicates (N = 3) **(C-D)** Characterisation of growth in a under experimental conditions. Large (25 ml) cultures were incubated in parallel, and their growth characterised at key time points by change in media color (ratio of OD at 430 and 560 nm) (C) and protein biomass (D). The samples shown here came from the same aliquot extracted from the culture. Colour change was measured with one technical replicate and two biological replicates (N = 2). Protein concentration was determined from two technical replicates and two biological ones (N = 2). Data is plotted as an average +/- the SD.


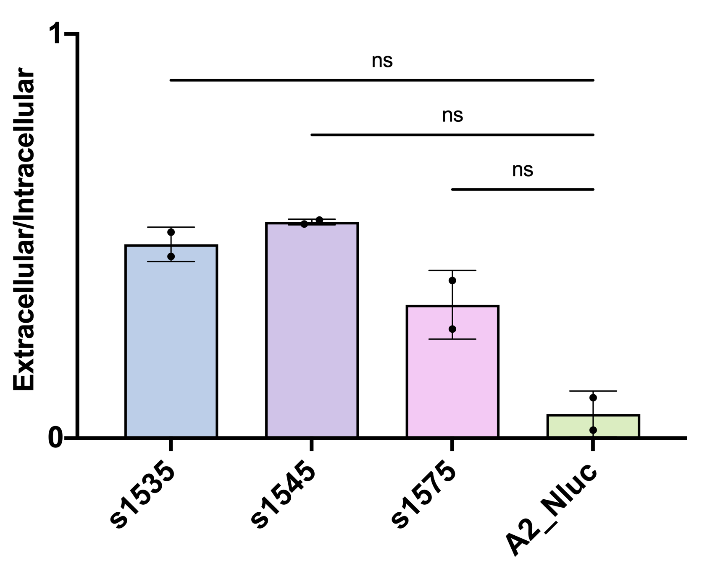


**Supplementary Figure 3. Secretion efficiency of signal peptides belonging to the MIB-MIP system.**  *Mfr* strains expressing signal peptides s1535, s1545 and 1575 coupled to a Nluc luminescent reporter were grown to exponential phase. Luminescence of both supernatant (extracellular) and pellet (intracellular) phases was measured to obtain a secretion efficiency ratio. Data are shown as average +/- SD of two biological and technical replicates (N = 2). Statistical analysis was performed using One-way ANOVA test of multiple comparisons.


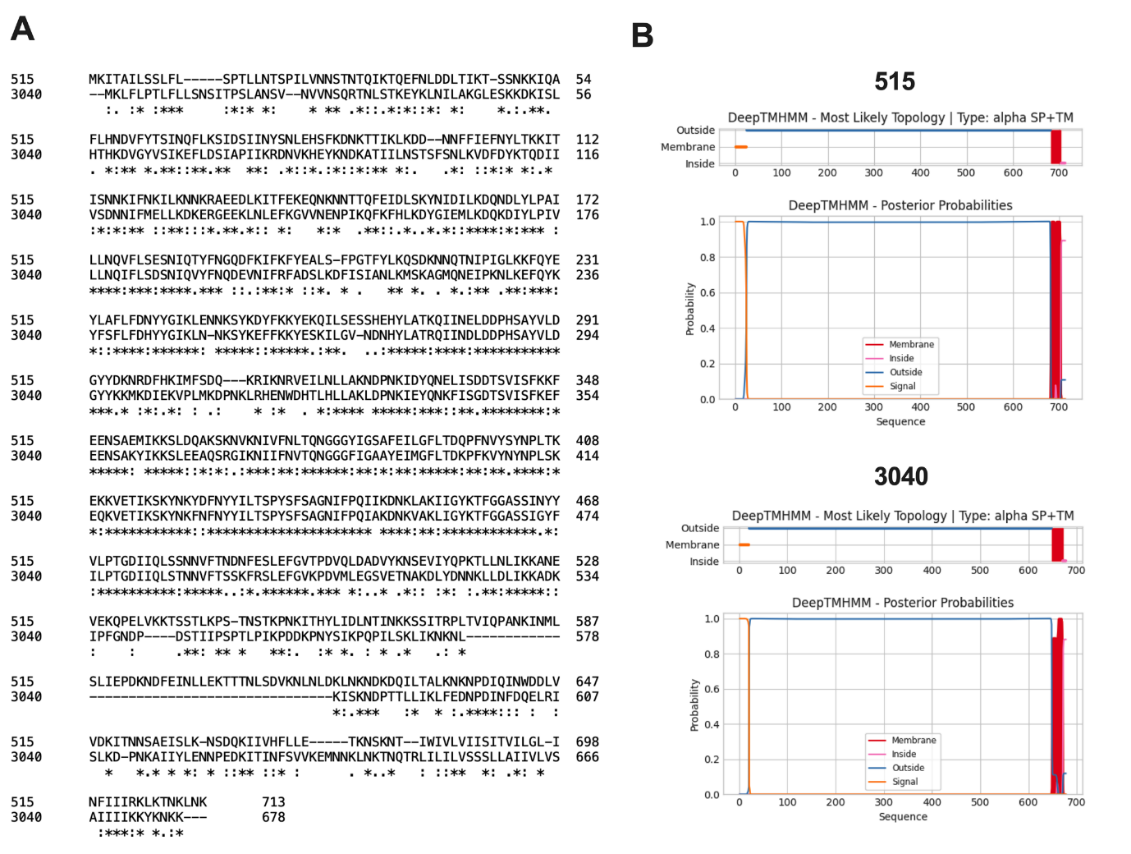


**Supplementary Figure 4.** **(A)** Alignment of the 515 and 3040 proteins using ClustalW server (https://www.ebi.ac.uk/Tools/msa/clustalo/). **(B)** Protein structure of 515 and 3040 determined by DeepTMHMM server (42). Both molecules have similar topology, where a signal peptide (orange) carries a protein to the surface leading (blue) to an extracellular domain that is anchored at the membrane by a C-terminal transmembrane domain (red).
